# Cross-talk between cd1d-restricted nkt cells and γδ cells in t regulatory cell response

**DOI:** 10.1186/1743-422X-8-32

**Published:** 2011-01-21

**Authors:** Wei Liu, Sally A Huber

**Affiliations:** 1 The First Affiliated Hospital of Harbin Medical University, Harbin,150001, PR China; 2 University of Vermont, Burlington, VT, 05405, USA

## Abstract

CD1d is a non-classical major histocompatibility class 1-like molecule which primarily presents either microbial or endogenous glycolipid antigens to T cells involved in innate immunity. Natural killer T (NKT) cells and a subpopulation of γδ T cells expressing the Vγ4 T cell receptor (TCR) recognize CD1d. NKT and Vγ4 T cells function in the innate immune response via rapid activation subsequent to infection and secrete large quantities of cytokines that both help control infection and modulate the developing adaptive immune response. T regulatory cells represent one cell population impacted by both NKT and Vγ4 T cells. This review discusses the evidence that NKT cells promote T regulatory cell activation both through direct interaction of NKT cell and dendritic cells and through NKT cell secretion of large amounts of TGFβ, IL-10 and IL-2. Recent studies have shown that CD1d-restricted Vγ4 T cells, in contrast to NKT cells, selectively kill T regulatory cells through a caspase-dependent mechanism. Vγ4 T cell elimination of the T regulatory cell population allows activation of autoimmune CD8+ effector cells leading to severe cardiac injury in a coxsackievirus B3 (CVB3) myocarditis model in mice. CD1d-restricted immunity can therefore lead to either immunosuppression or autoimmunity depending upon the type of innate effector dominating during the infection.

## Introduction

Myocarditis is an inflammation of myocardium with subsequent cardiomyocyte death, replacement fibrosis, and cardiac dysfunction [1,2], is a significant cause of sudden death in children and young adults [3-7], and often follows cardiac infections (virus, bacteria, fungus, worms) [8]. Enteroviruses and adenoviruses cause approximately 80% of clinical viral myocarditis with human cytomegalovirus, parvovirus, influenza virus, and herpes simplex virus infection causing most of the remainder [9]. Cardiac injury results from direct viral injury to infected cardiocytes and from the host response to infection[10]. Strong evidence exists for immunopathogenic mechanisms of cardiac injury in experimental models of coxsackievirus B3 (CVB3) induced myocarditis. T cell depletion of mice dramatically reduces animal mortality and cardiac inflammation [11], and heart-specific, autoimmune CD8+ T cells isolated from CVB3 infected mice [12] transfer myocarditis into uninfected recipients. A major question is how the virus activates these autoimmune CD8+ T cells. Antigenic mimicry between CVB3 and cardiac myosin forms the basis for the autoimmunity [13,14]. However, some CVB3 variants replicate in the heart but fail to activate autoimmunity [15]. The crucial difference between the variants is that the pathogenic virus induces CD1d up-regulation on hemopoietic and non-hemopoietic cells but the non-pathogenic variant does not [16-18], and this failure to up-regulate CD1d leads to generation of T regulatory cells [19].

### CD1 molecules and regulation of their expression

CD1 molecules belong to a family of non-polymorphic, class I-like major histocompatibility complex (MHC) molecules, which bind and present amphiphilic lipid antigens to T cells for recognition [20]. The CD1 family in humans and most other species are divided into transmembrane Group 1 (CD1a,b,c), and Group 2 (CD1d) molecules [21,22]. An intermediate isoform (CD1e) exists as a soluble molecule in the late endosome where it facilitates processing of complex glycolipids for presentation by other CD1 isoforms [23]. Group 1 CD1 molecules are expressed on thymocytes, dendritic cells, activated monocytes and B lymphocytes. CD1d is expressed on these cells and additionally on T cells and non-hemopoietic cells including cardiac myocytes and endothelial cells [16,22,24]. While structurally similar to class I MHC molecules (consisting of a single polypeptide chain coded by the CD1 gene and associated with β2 microglobulin), antigen presentation resembles class II MHC molecules since antigen loading occurs in the endosome pathway and is TAP independent [25-27]. The CD1 extracellular domain contains a deep antigen binding groove comprised of up to four hydrophobic pockets into which lipid tails of antigens are inserted [28-30]. CD1b presents bacterial lipids including mycobacterial mycolic acids [31], lipoarabinomannan [32], glucose monomycolate [33], and self-glycosphingolipids such as GM1 ganglioside [34]. CD1a and CD1c present bacterial phospholipids [35]. CD1d presents a bacterial sphingolipid from *Sphingomonas *[36], alphaproteobacterium from *N. aromaticivorans *[37], glycolipids from *B. burgdorferi *[38], and a self-sphingolipid isogloboside [39]. The sphingolipid α-galactosylceramide (αGalCer) isolated from marine sponges, is the classical CD1d ligand for activating NKT cells [40]. CD1 molecules also bind and present other endogenous (self) glycolipid sulfatides [41-44]. Lysosomal α-galactosidase A is highly effective in degrading endogenous lipid antigens, normally limiting autoreactive NKT cell responses [44]. However, infections inhibit α-galactosidase A activity allowing endogenous lipid accumulation and NKT cell activation. This means that CD1d dependent innate immunity may be directed to both exogenous and endogenous antigens during infections.

Group 1 CD1 molecules are not constitutively expressed on myeloid precursors of dendritic cells, but can be induced by signaling through TLR2 [45], TLR2/TLR5 agonists, or cytokines (GM-CSF and IL-4) during differentiation into immature dendritic cells [46]. CD1d is constitutively expressed in dendritic cells at most stages of differentiation, as well as on monocytes and macrophage [47]. Unlike Group 1 CD1 molecules, CD1d is not up-regulated by GM-CSF and IL-4 [24,48], but is up-regulated by exposure to bacteria or viruses [16,49,50]. Studies using *M. tuberculosis *found that both signaling through TLR2 and cytokines (IFNγ and TNFα) were required for CD1d up-regulation on macrophage both in vitro and in vivo [51]. Similarly, studies using *L. monocytogenes *found that IFNβ increases CD1d expression [52]. CVB3 infection augments CD1d expression on macrophage, dendritic cells and T cells [18]. The virus also causes de novo CD1d expression on non-hemopoietic cells (cardiac endothelial cells and myocytes), but only in non-hemopoietic cells actively replicating virus. Immediately adjacent uninfected myocytes/endothelial cells remain CD1d negative [16]. CD1d expression requires TNFα, but TNFα treatment of uninfected endothelial cells alone cannot induce CD1d [53], indicating that a separate signal besides cytokine exposure is necessary for de novo CD1d synthesis and that this signal must be generated through direct virus-cell interactions. In fact, CVB3 binding to decay accelerating factor (DAF) one of the two know cellular receptors for [54], provides the essential secondary signal [17]. DAF is a glycosylphosphatidylinositol (GPI)-anchored membrane glycoprotein and its primary biological function is to prevent autologous complement induced cell injury by inactivating C3 [55-57]. Signaling through this molecule by C3 or CVB3 induces calcium flux and activation of the transcription factor, NFAT [58,59]. CVB3 infection of DAF deficient cells fails to induce CD1d expression [17], and blocking NFAT activation either by use of cells expressing a dominant-negative-NFAT or by cyclosporine A prevents CD1d expression in virus infected cells. Thus, only cells which are exposed to TNFα and bind virus to DAF on the cell membrane will up-regulate CD1d.

While many microbial infections augment CD1 expression, other infectious agents suppress expression of these molecules on dendritic cells and antigen presenting cells [60-62]. In Leishmania, cytomegalovirus, and herpes simplex virus infections, CD1 expression is down-regulated. With HSV, modulation of CD1 was dependent upon the amount of virus with low levels of virus enhancing both type 1 and 2 CD1 expression on human dendritic cells while high levels of virus suppressed CD1 expression. In HSV, suppression correlated with accumulation of intracellular viral protein and interruption of CD1 recycling pathway. However, other studies demonstrate that activating TLR7/8, TLR recognizing single strand RNA and RNA viruses, block CD1 expression at the protein and mRNA levels [63]. Where CD1-restricted immunity promotes host defense against infection, inhibition of CD1 up-regulation can provide an evasion mechanism for the microbes.

### Role of CD1d in Innate Immunity and Infection

CD1d-dependent innate immunity is important in a wide range of diseases. Infections with *P. aeruginosa *[64], *C. neoformans *[65], Herpes Simplex virus [66], Hepatitis C virus [50], and encephalomyocarditis virus [67,68] make the disease substantially worse in animals lacking CD1 or NKT cells. In contrast, clearance of RSV is delayed in CD1d deficient mice [49,69]. CD1-restricted cells appear to have minimal effects in cytomegalovirus and lymphocytic choriomeningitis virus infections [70]. In Novosphingobium aromaticivorans infection in mice, CD1d presentation of alpha-glycuronosylceramide from the bacterial cell wall activates NKT cells and ultimately results in liver-specific autoimmunity [37]. This means that CD1d-restricted effectors may either have no, beneficial or detrimental functions depending upon the infectious agent.

There are two major populations of CD1-restricted T cells. These are NKT cells and γδ T cells. NKT cells co-express T cell receptors (TCR) and NK receptors. There are two types of NKT cells. Type 1 NKT cells have a TCR comprised of a single type of TCRα chain (Vα14Jα18 for mice and Vα24Jα18 for humans) and one of a limited number of distinct TCRβ chains resulting in limited clonal diversity. These cells are usually designated as invariant NKT (iNKT) cells. Type 2 NKT cells use diverse TCR (non-Vα14Jα18/Vα24Jα18). Both Type 1 and Type2 NKT cells are CD1d restricted [71-73]. NKT cells comprise up to 2% of spleen, 20% of mononuclear cells in the liver and 40% of CD3+ cells in bone marrow in the mouse making these cells a major component of the total T cell population [74,75]. NKT cells have a constitutively activated phenotype and are capable of rapidly secreting large amounts of cytokines (IFN-γ, IL-4, IL-17, IL-5, and IL-13) upon activation, which can modulate many immunological processes, including tumor immunity, maintenance of immunologic self tolerance, prevention of autoimmune disorders, and protection from a variety of pathogens during experimental infections [73,76,77]. Rapid cytokine secretion occurs because cytokine mRNAs pre-exist in the cells [78]. The presence of pro-inflammatory cytokines (IL-12 and IL-12/IL-18) can dramatically reduce the amount of CD1-dependent TCR signaling required for NKT cell activation [79,80]. Type 1 NKT cells produce IFNγ and IL-2 which activates NK cells and dendritic cells, enhancing antigen presentation [81,82]. Rapid cytokine secretion by the NKT cells polarizes developing adaptive immunity along the Th1/Th2 axis [83]. Both Type 1 and Type 2 NKT cells can have a Th1 or Th2 phenotype with corresponding cytokine profiles, and therefore may have either potentiating or protective roles in infections and autoimmune diseases [84]. The majority of reports indicate that Type 2 NKT cells are protective in autoimmune diseases in mice including in models of autoimmune diabetes in NOD mice [85], EAE [86] and Con-A induced hepatitis [87]. Furthermore, while type 1 NKT cells may increase tumor immunosurveillance, type 2 NKT cells may suppress anti-tumor immunity [74,88].

NKT cells are not the only CD1 restricted lymphocyte. Human γδ T cells recognize lipid antigens presented by CD1 [89,90], and mouse γδ T cells recognize non-classical MHC antigens (T10/T22) [91,92]. γδ cells expressing the Vγ4 TCR recognize CD1d [16]. Within 2-4 days of CVB3 infection, CD1d is rapidly up-regulated on cardiac endothelial cells and myocytes and results in infiltration of Vγ4+ T cells into the myocardium [93]. As with NKT cells, Vγ4+ cells rapidly secrete large amounts of pro-inflammatory cytokines including TNFα and IFNγ which establish an environment conducive to polarizing the developing virus specific adaptive immune response to a Th1 phenotype [94,95]. The Vγ4+ cells also kill CVB3 infected CD1d+ cardiocytes in a Fas-dependent manner which aids in viral control by eliminating infected cells early in the virus replication cycle. As with NKT cells, γδ cells can interact with CD1 on dendritic cells/macrophage resulting in enhanced antigen presentation and cytokine release [96,97]. CVB3 infection of mice lacking γδ T cells results in increased virus titers in the heart but little or no cardiac inflammation, animal mortality, or heart-specific autoimmunity[98-100] making γδ cells essential in the pathogenesis of CVB3 infections.

Several cases of clinical cardiomyopathy where γδ cells dominate the inflammatory infiltrate have been reported [101-103] suggesting that these innate effectors can be directly pathogenic. More often, γδ cells would impact myocarditis through their effects on the antigen-specific, adaptive immune response. In this laboratory's mouse model of CVB3 induced myocarditis, infection activates heart-specific, autoimmune CD8+ cytolytic T lymphocytes [12,100,104,105] which kill uninfected cardiocytes through recognition of cardiac myosin epitopes [14] and can adoptively transfer myocarditis into uninfected recipients [106]. These autoimmune CD8+ effectors are the primary cause of cardiac injury. In vivo generation of autoimmune CD8+ cells requires activated Vγ4 cells and mice lacking either the Vγ4 or all γδ cells do not generate autoimmunity [100]. Thus, the primary role of Vγ4 cells in CVB3 induced myocarditis is to facilitate autoimmunity induction.

### Role of CD1d in CD4+CD25+ regulatory T cell response

Regulatory T cells (Tregs) are important negative immune modulators, constitute up to 10% of peripheral CD4+ T cells in naive mice and humans, and express CD25 (IL-2 receptor α chain) [107-109]. Several subsets of T regulatory cells have been described and these can basically be divided into natural (nTreg) and inducible (iTreg) populations. The nTreg cells arise in the thymus during normal T cell ontogeny as CD4+CD25+ cells and depend upon expression of the FoxP3 transcription factor. Indeed FoxP3 expression is crucial to the immunosuppression activity of these nTreg since transduction of exogenous FoxP3 into CD4+CD25- cells is sufficient to convert these cells into CD4+CD25+ Treg cells [108]. Developing T cells with high affinity TCR for self antigens are most probably committed to the nTreg line. While most αβ TCR+ cells (exclusive of NKT cells) developing in the thymus enter the periphery as naïve cells, nTreg cells are functionally mature when leaving the thymus and do not require antigen exposure peripherally to generate immunosuppressive activity. While FoxP3 is necessary for conversion of CD4+ cells to Treg cells, IL-2 is required for Treg cell maintenance/survival. Animals lacking either CD25 (IL-2R) or IL-2 develop lymphoproliferative and autoimmune diseases [110] associated with a decrease in Treg cells. Although the transcription factor NFAT normally increases expression of IL-2and IFNγ while decreasing expression of CD25 and CTLA4, NFAT complexed with FoxP3 has the opposite effect, decreasing IL-2/IFNγ and increasing CD25/CTLA4 expression. In addition to nTreg cells, inducible regulatory T cells (iTreg) can be converted from effector T cell populations in the periphery subsequent to antigen challenge. These iTreg cells are CD4+CD25+ but can either be FoxP3+ or FoxP3- [111]. Both iTreg and nTreg cells can secret either IL-10 (Tr1) or TGFβ (Th3) cytokines [111]. Treg cells express similar chemokine receptor patterns as effector T cells and can migrate to peripheral lymphoid tissues and inflammatory sites similarly to the effector population [108]. There are three general hypothesized mechanisms for Treg cell suppression of immunity [108]. First, T reg cells may bind to and out-compete effector T cells for MHC-antigen complexes on dendritic cells and effectively block antigen presentation to the effector T cell populations. Secondly, direct Treg-dendritic cell interactions through CTLA4 can down-regulate accessory molecule expression (CD80/CD86) on the dendritic cells making them less effective in antigen presentation. Third, Treg cells can either kill or inhibit T cell differentiation. TGFβ produced by Treg cells will activate NOTCH and its downstream target gene, Hes1, which suppresses gene expression in T cells [111]. IL-10 blocks CD2, CD28 and ICOS signaling in T cells and SOCS3 signaling in monocytes resulting in reduced T cell proliferation and cytokine response [112].

Treg cells play an important role in preventing autoimmunity in myocarditis [19,113,114]. Two CVB3 variants have been identified which differ by a single non-conserved mutation in the VP2 capsid protein in a region associated with DAF binding [15]. One variant, designated H3, binds with high avidity to DAF, causes calcium flux and NFAT activation, induces CD1d expression in the heart and activates Vγ4 cells [16,17,19,59]. The other variant, designated H310A1, binds with low affinity to DAF, fails to activate NFAT, does not up-regulate CD1d expression in the heart, and does not activate Vγ4 cells. While H3 virus induces autoimmune CD8 T cells and causes severe myocarditis, the H310A1 virus fails to induce autoimmunity and induces minimal cardiac injury despite high virus titers in the heart [115]. The primary difference between the two virus infections is that H310A1 infection activates CD4+CD25+FoxP3+ Treg cells which are absent in H3 infected mice [19].

Innate effector T cells control Treg cell responses. Although somewhat controversial, various reports indicate that iNKT cells suppress autoimmunity by promoting T regulatory cell activation. Studies investigating oral tolerance to nickel demonstrated that antigen presenting cells interact with type 1 NKT cells through CD1d causing the NKT cells to secrete IL-4 and IL-10 and activate Treg cells [116-118]. Similar studies found that T regulatory cells fail to generate in CD1dKO mice [119] and iNKT KO mice [119]. Other studies show that αGalCer, a well known and specific NKT CD1d-restricted ligand, increases Treg cell numbers in vivo [120] and can suppress autoimmune diabetes in NOD mice [121-123]. NKT cells secret high levels of TGFβ and IL-10 [124,125] which alter dendritic cell (DC) cytokine (IL-10) and accessory molecule (CD40, CD80 and/or CD86) expression [126-128] that favors T regulatory cell responses [129,130].

A number of reports indicate that γδ T cells can also affect Treg cell responses. IL-23 activated γδ cells prevent conversion of effector T cells to iTreg cells [131]. Similarly γδ cells reduce IL-10 producing Treg cells in the lung in an asthma model γδ cells [132]. Vγ2Vδ2 cells prevent IL-2 induced expansion of CD4+CD25+FoxP3+ T [133]. Therefore, while many studies suggest that NKT cells promote Treg cell activation and protect from autoimmunity, it appears that γδ T cells can have the opposite effect and promote autoimmunity/inflammation through inhibiting T regulatory cell activity. As with NKT cells, the mechanism by which γδ T cells modulate Treg cell responses can be diverse and include alterations in antigen presenting cells which prevent Treg cell activation, and suppression of IL2 needed for Treg cell maintenance. This laboratory has recently reported an additional mechanism for γδ T cell modulation of Treg cell responses using two coxsackievirus B3 variants which differ by a single non-conserved amino acid in the VP2 capsid protein [15]. These studies show that the non-pathogenic variant induces a potent T regulatory cell (CD4+CD25+FoxP3+) response which is absent during infections with the pathogenic virus [19]. Although H3 infected mice normally have few CD4+CD25+FoxP3+ cells, H3 infection of γδ cell deficient mice results in significant increases in Treg cells and suppression of myocarditis. Therefore, this study, as others mentioned above, find that γδ cells antagonize Treg cell responses and promote autoimmunity. Further studies demonstrate that a subpopulation of Treg cells in γδ deficient mice express high levels of CD1d, that the CD1d+ Treg cells are substantially more immunosuppressive on a per cell basis than CD1d- Treg cells, and that Vγ4+ cells selectively kill the CD1d+ Treg cells in a CD1d and caspase-dependent manner [10].

## Conclusions

**Figure 1:**
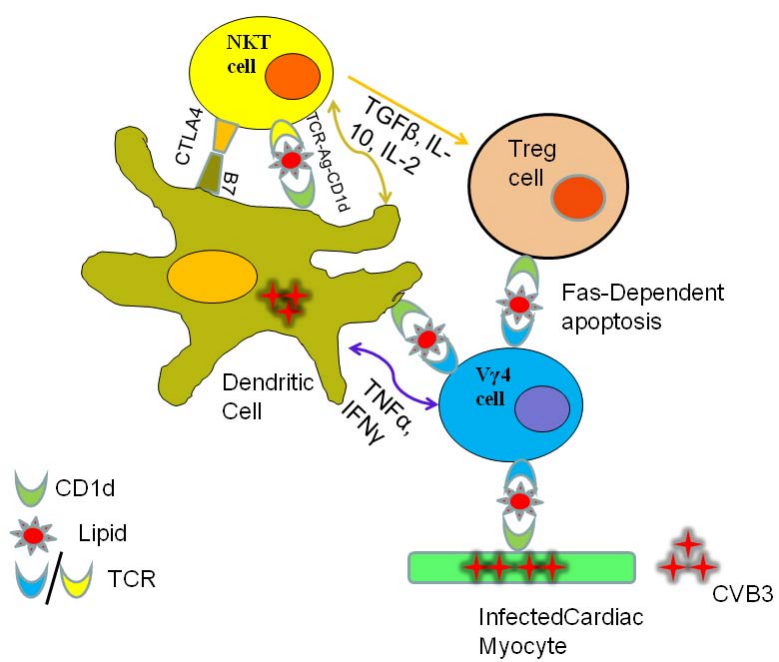
**CVB3 up-regulates CD1d on dendritic cells and non-hemopoietic cells such as cardiac myocytes**. CD1d-glycolipid complexes on dendritic cells activate NKT and Vγ4+ T cells which kill virus infected cells limit virus infections, while the activated NKT/Vγ4+ cells also alter dendritic cells. CTLR4 expression by NKT cells either blocks or down-regulates B7 co-stimulatory molecules inhibiting antigen presentation to antigen-specific CD4+ and CD8+ T cells, and promoting inducible Tregulatory cell activation through release of TGFβ, IL-10 and IL-2. TNFα and IFNγ secretion by Vγ4+ T cells enhances maturation and antigen presentation by dendritic cells but suppresses activation of the inducible Tregulatory cell. CVB3 infection up-regulates CD1d expression on a subpopulation of CD4+CD25+FoxP3+ Tregulatory cells. The CD1d+Tregulatory cells are substantially more immunosuppressive than CD1d- Tregulatory cells and are primarily responsible for preventing autoimmunity to cardiac antigens and myocarditis during CVB3 infection. Vγ4+ T cells selectively kill CD1d+ T regulatory cells through caspase-dependent apoptosis which then results in autoimmunity induction. Thus, pathogenesis in CVB3 infection depends upon the balance in NKT and Vγ4+ T cell activation.

Innate immunity is crucial to anti-microbial host defense as it helps control infectious agents until a more potent microbe-specific, adaptive immune response can be generated. However, the innate response is also important in molding the nature of the adaptive immune response. CD1 molecules, as members of the non-classical MHC family, are intimately involved in innate immunity. NKT and a subset of γδ T cells are CD1 restricted. Figure 1 provides a schematic of the interactions between innate effectors and the adaptive immune response through CD1d. This raises the interesting question of why two innate effector populations would exist which respond to the same type of antigen presenting molecule, especially since CD1 molecules have limited diversity and are therefore more likely to present less heterogeneous antigens than classical MHC antigens. Two possibilities are that CD1-restricted NKT and γδ T cells have redundant functions or that each type of CD1-restricted effector has a distinct role in the immune response. One potential reason for redundancy is that NKT and γδ cells tend to concentrate in different tissues. NKT cells comprise 20-30% of liver and bone marrow T cells but are generally absent in intestinal epithelial lymphocytes (IEL) [134]. In contrast, γδ concentrate in epithelia of skin, intestine and reproductive tract where these cells can comprise up to 50% of the T cells [135]. This distribution could imply that CD1 presentation of microbial or self antigens will preferentially activate NKT or γδ cells depending upon the tissue involved. In peripheral lymphoid organs where both NKT and γδ cells are present, other factors must determine if CD1-dependent NKT or γδ cell responses predominate. What these factors are is not known. One possibility is that while CD1 presents a variety of glycolipid antigens, NKT and γδ cells recognize distinct sets of these antigens. In this case, CD1d might exclusively activate only NKT or γδ cells depending upon which glycolipid antigens are presented. A second possibility is that the binding avidity for CD1d-antigen complexes differ for NKT and γδ cells meaning that the innate effector with the stronger binding avidity would dominate. The relevant point is that the balance between NKT and γδ activation can be the deciding factor between self-tolerance and autoimmunity. The major unresolved question is what decides whether γδ or NKT cells dominate in an innate response where CD1 is up-regulated.

## Conflict of interest statement

The author states that they have no conflict of interest.

## Authors' contributions

WL intellectually summarized the role of CD1d in virus induced myocarditis. She participated in the experiment (NKT related) and was involved in drafting the manuscript. SAH was involved in most of the basic experiments being summarized in this review. She designed most of the experiments we mentioned and revised this manuscript. Both authors read and approved the final manuscript.
